# Myogenic differentiation and reparative activity of stromal cells derived from pericardial adipose in comparison to subcutaneous origin

**DOI:** 10.1186/scrt481

**Published:** 2014-08-01

**Authors:** Xiaoming Wang, Hui Zhang, Liangming Nie, Linhai Xu, Min Chen, Zhaoping Ding

**Affiliations:** Department of Cardiothoracic Surgery, Zhejiang Provincial People’s Hospital, Hangzhou, China; Operation Theatre, Zhejiang Provincial People’s Hospital, Hangzhou, China; School of Medical Science and Laboratory Medicine, Jiangsu University, 301 XueFu Road, Zhenjiang, 212013 P.R. China; Institute of Molecular Cardiology, Heinrich-Heine University of Düsseldorf, Moorenstr 5, Düsseldorf, 40225 Germany

## Abstract

**Introduction:**

Adipose tissue-derived stromal cells (ADSCs) are abundant and easy to obtain, but the diversity of differentiation potential from different locations may vary with the developmental origin of their mesenchymal compartment. We therefore aim to compare the myogenic differentiation and reparative activity of ADSCs derived from the pericardial tissue to ADSCs of subcutaneous origin.

**Methods:**

Pericardial and inguinal adipose tissues from Wistar rats were surgically obtained, and the stromal fraction was isolated after enzymatic digestion. The phenotypic epitopes of the resultant two types of ADSCs were analyzed with flow cytometry, and the expression of transcriptional factors was analyzed with immunostaining. Furthermore, their potential toward adipogenic, osteogenic, and myogenic differentiation also was compared. Finally, the reparative activity and the resultant functional benefits were examined by allograft transplantation into an infarcted model in rats.

**Results:**

ADSCs from two adipose sources showed identical morphology and growth curve at the initial stage, but inguinal ADSCs (ingADSCs) sustained significantly vigorous growth after 25 days of cultivation. Although both ADSCs shared similar immunophenotypes, the pericardial ADSCs (periADSC) intrinsically exhibited partial expression of transcription factors for cardiogenesis (such as GATA-4, Isl-1, Nkx 2.5, and MEF-2c) and more-efficient myogenic differentiation, but less competent for adipogenic and osteogenic differentiation. After *in vivo* transplantation, periADSCs exhibited significantly vigorous reparative activity evidenced by thickening of ventricular wall and pronounced vasculogenesis and myogenesis, although the majority of prelabeled cells disappeared 28 days after transplantation. The structural repair also translated into functional benefits of hearts after infarction.

**Conclusions:**

Although two sources of ADSCs are phenotypically identical, pericADSCs constituted intrinsic properties toward myogenesis and vasculogenesis, and thus provided more potent reparative effects after transplantation; therefore, they represent an attractive candidate cell donor for cardiac therapy.

## Introduction

Adipose tissue is now recognized as an abundant, easily accessible source for obtaining regenerative stromal/mesenchymal stem cells (MSCs) [1]. Adipose-derived stem cells (ADSCs) are a highly proliferative cell population that has the potential to differentiate toward multiple mesodermal lineages, such as cardiomyocytes, cartilage, and osteocytes in optimal conditions [2]. Therefore, ADSCs have been recently made use of tissue engineering and stem cell-based therapy in both experimental settings and clinic trails [3, 4].

Although it has been assumed that all MSCs from different adipose tissues are phenotypically similar and homogeneous, this notion ignores important biologic diversity among various ADSCs [5]. The cell source-related heterogeneity may not only be related to cell proliferation, clonogenicity, and the potential to differentiate, but also may greatly affect the survivability and behavior after being transplanted into the hostile environment of ischemia, inflammation, proapoptotic factors, and scarring from myocardial infarction [3, 6]. Recently, Naftali-Shani *et al*. [6] systematically compared the cellular epitopes, cytokine-secretion profiles, and immunomodulatory properties of MSCs from five different origins of cardiac patients and suggested that ADSCs from different locations have both distinct characteristics and different reparative properties [6]. This result also raises the question whether the ADSCs from different sources share a similar potential toward myogenic differentiation, a key issue for cardiac regeneration.

In the view of developmental biology, adipose tissue originates from mesoderm, but the tissue stromal cells are highly homologous with the organs in which they reside, leading to biologic variations, including the differentiation potency toward certain cell types [7]. In heart, the epicardial and pericardial ADSCs were formed during embryonic heart development from the neural crest and the pro-epicardial organ (a cluster of cells located dorsal and adjacent to the looped heart tube) that migrated onto the surface layer of the heart tube to form the second heart field, including the coronary system, epicardium, and pericardium, as well as subepicardial myocardium [8]. The pericardium is a continuous layer of parietal epicardium and consists of a thin fibrous layer, nerves and vascular network, and adipose mass. The pericardial tissue, analogous to the epicardium, has been recently shown to comprise a mesodermal origin of mesenchymal cells, so-called formed cardiac colony-forming-unit fibroblasts (cCFU-Fs) that give rise to all mesodermal lineages, including smooth muscle, bone, cartilage, adipose, endothelial, and heart muscle cells [9].

Therefore, we hypothesized that pericardial fat could provide stromal stem cells with better cardiomyogenic differentiation and reparative activity because of their proximity to the heart. In the present study, we compared the immunophenotypes and the differential efficiency of pericardium-derived ADSCs with the ADSCs from the extracardiac subcutaneous fat tissues that are nowadays widely used in clinical trials [3]. Our results showed that the pericardial ADSCs are more competent for myogenic differentiation and yielded more-efficient cardiac repair *in vivo*. Because pericardial fat tissue can be surgically obtained, pericardial ADSCs represent an attractive cell source for stem cell-based therapy for ischemic heart disease.

## Methods

### Isolation and cultivation of ADSCs

All the experiments were approved by the Experimental Animal Care and Use Committee at the Zhejiang Chinese Medical University (SCXK Hu 2008–0016). The ADSCs were isolated according to the modified protocol previously reported [3]. In brief, Wistar male rats with body weight of 200 to 300 g (*n* = 29) were killed by using CO_2_ suffocation followed by immersion with 75% ethanol for 3 minutes for the entire body disinfection. Under sterilized conditions, rat chest and the lower abdomen were cut to harvest inguinal and pericardial adipose tissues. The collected adipose tissues were first cut into small pieces and washed with PBS to remove adipose drops. Then the tissue pieces were mixed with 10 ml of collagenase (0.4%, Biochrom, Germany) and incubated at 37°C with gentle rotation (20 rpm) for 20 minutes. The activity of the collagenase was neutralized with DMEM medium containing 30% FCS (HyClone, USA), and the cell suspension was spun down at 1,000 rpm for 5 minutes. The supernatant was then discarded, and the cell pellet was resuspended with basic medium containing low-sugar DMEM and supplemented with 30% FCS, penicillin (100 U/ml), streptomycin (0.1 mg/ml), and glutamine (2 m*M*). The isolated cells were inoculated in a density of 2 × 10^3^/cm^2^ into a T25 culture flask and incubated at 37°C with 5% CO_2_. Nonadherent cells were removed 12 hours after initial plating by intensely washing the flask, and the resulting adherent stromal vascular fraction was then termed adipose-derived stromal cells (ADSCs). Both pericardial and inguinal ADSCs were cultivated under the same conditions and harvested at subconfluence by using 0.05% trypsin-EDTA (Sigma). Cell numbers were counted in a small aliquot on 5, 8, 11, 15, 25, 50, and 70 days, respectively, and the total cell numbers were calculated for plotting cell-growth curves.

For genetic labeling of periADSCs with eGFP, subconfluent periADSCs were transfected with a lentiviral vector derived from the HIV1-vector pGJ3-CSCGW, carrying eGFP under the control of the U3 promoter of spleen focus-forming virus (SFFV). After infection, periADSCs were exhaustively washed (>10 times with PBS), and the expression of eGFP was examined with fluorescence microscopy and qualified with flow cytometry.

### Characterization of the surface epitopes for the two sources of ADSCs

For the characterization of the surface epitopes of the isolated ADSCs, the cultured ADSCs were allowed to grow up to 90% confluence and analyzed with fluorescence-activated flow cytometry (*n* = 4 for periADSCs and *n* =3 for ingADSCs, all first passage). The following cell-surface epitopes were marked with anti-murine antibodies: IgG1, CD29, CD31, CD34, CD44, CD45, CD90 (Becton Dickinson, USA), and CD106 (Miltenyi Biotec, Germany). Cells were incubated with antibodies with a dilution of 1:100 to 200 for 30 minutes at room temperature. After washing 3 times, we analyzed about 1,000 labeled cells analyzed by using a FACScan flow cytometer running CellQuest software (Becton Dickinson, USA).

### Immunocytochemistry

For immunocytochemical analysis, the primary cells (first passage) grown in chamber slides were fixed with 1% paraformaldehyde and permeabilized with 0.25% Triton for 30 minutes. After 1 hour of blocking in 5% normal goat serum (NGS), cells were incubated for 1 hour with primary antibodies, including polyclonal anti-cTnT (DAKO, Germany) 1:100 and polyclonal anti-GATA-4, isl-1, Nkx-2.5, and MEF-2c 1:50 (Santa Cruz, USA). After washing in 1% NGS-PBS buffer, we added secondary TRTIC-conjugate antibody (goat IgG, Santa Cruz) and incubated for 60 minutes. Nuclei were counterstained with DAPI (DAKO), and the chamber slides were sealed with Prolong Gold (Life Science, USA). All visualization was digitalized by using phase-contrast microscopy (Olympus BX61, Japan) with software (SIS F-View).

### Induction of myogenic, adipogenic, and osteogenic differentiation

To induce ADSCs to adipogenic differentiation, both periADSCs and ingADSCs at passage 2 were seeded at a density of 2 × 10^4^ cells/cm^2^ in basal medium. After 24 hours, medium was switched to high-glucose adipogenic induction medium (Gibco, life technologies, USA), supplemented with 10% FCS, 1% L-glut, 1% Pen-Strep, 1 μ*M* dexamethasone, 1 μ*M* indomethacin, 500 μ*M* 3-isobutyl-1-methylxanthine (IBMX, Sigma, USA), and 10 μg/ml human recombinant insulin, as previously reported [1]. The culture medium was changed twice a week, and induction was maintained for 2 weeks before oil red staining was performed.

To promote osteogenic differentiation, the culture medium was switched to osteogenic medium (Cyagen, China), containing 10% FCS, 1% Pen-Strep, 1 μ*M* dexamethasone), 50 μ*M* ascorbate, and 10 μ*M* β-glycerophosphate (Sigma, USA) [1]. The culture medium was changed twice per week, and induction was maintained for 2 weeks before alizarin red staining was performed, as described later.

For the induction of ADSCs toward myogenic differentiation, both periADSCs and ingADSCs were seeded at a density of 3.1 × 10^3^ cells per cm^2^ into eight-chamber slides (Nunc, Thermo Scientific, USA) and cultured in DMEM culture medium. As soon as subconfluence was reached, cardiac differentiation of the ADSCs was induced by changing the culture medium into DMEM supplemented with only 5% FCS, 5% horse serum together, with 0.1 m*M* 5-azacytidine (Sigma) and 0.1 μ*M* dexamethasone (Sigma). Medium was changed every 3 days up to 2 weeks, until cultures were fixed and stained for cardiac troponin T (cTnT) for assessment of cardiomyogenic differentiation. Percentage was derived by counting the number of cTnT-positive cell versus total cell numbers (DAPI positive).

### *In vivo*allograft transplantation

To compare directly the regenerative properties of different sources of ADSCs, we injected the two types of ADSCs into the rat heart after 60 minutes of ischemia, as previously described [10]. In brief, male Wistar rats (250 to 320 g; *n* = 16) were intubated and anesthetized by mechanical ventilation with isoflurane (1.5% vol/vol; Abbott) in 100% oxygen and placed in a supine position with paws taped for ECG measurement. The chest was then opened with a lateral cut along the left side of the sternum. Ligation was done with a 6–0 polypropylene suture with a tapered needle passed underneath the LAD. The success of occlusion of the LAD was verified visually under the microscope by the absence of blood flow in the epicardium as well as significant elevations of the S-T segment. The occlusion was maintained for 60 minutes until the suture was released. Rats were randomized into two groups for periADSC (*n* = 5) or ingADSC (*n* = 5; one died of ischemia due to ventricular fibrillation) injection. Thereafter, two types of ADSCs at the second passage (500,000 in 50 μl PBS) were injected directly into a single spot at the middle of the infarcted ventricular free wall by using a 500-μl insulin syringe. Additionally, same amount of prelabeled periADSCs were applied into the infarcted hearts (*n* = 6) for the evaluation of the survival and integration of periADSCs into the host myocardium. After injection, a tiny drop of Trypan Blue (1 to 2 μl) was applied to mark the spot of injection. After the animal recovered from the transplantation procedure, the chest was closed with one layer through the muscle and a second layer through the skin. Animals then were weaned from ventilation and placed in a warm and oxygen-enriched environment until they fully recovered.

The survival of eGFP-periADSCs was analyzed in the early phase (1 hour, *n* =3) and long-term (28 days, *n* =3) by evaluation of GFP-positive cells in a total of 24 successive sections of the hearts. For the comparison of functional benefits 28 days after transplantation, the animals received either periADSCs (*n* = 5) or ingADSCs (*n* = 4) were anesthetized by inhalation of isoflurane (1.5% vol/vol) by using a home-made mask, and echocardiography (Hewlett Packard Sonos 5500 equipped with 15-MHz linear array probe, USA) was performed. All data were stored, and parameters of cardiac function were analyzed offline with customized software (Hewlett Packard). Thereafter, rats were killed, and the hearts were excised. After one washing in ice-cold PBS, the hearts were carefully positioned and embedded in Tissure-Tek (Leica, Germany) and snap-frozen in liquid nitrogen. Cryosections were made until the site of injection was defined (located about 5 mm from the apex of the heart). The successive 8-μm sections were made and fixed with paraformaldehyde for 30 minutes at room temperature. These heart sections were stained either with hematoxylin and eosin (H&E), Sirius Red, or indirect immunostaining of cTnT as a cardiac marker or vWF for an endothelial marker (see earlier). Photos were acquired by using a fluorescence microscope (MX 61, Olympus) and recorded by using a digital camera (FVreII and UC30; Olympus). The wall thickness was derived from five measurements at the site of injection that was marked with Trypan Blue in three successive sections with interval of 0.5 mm. The wall thickness and percentage of cardiomyocytes (cTnT positive) were analyzed in all animals (*n* = 5 in the periADSC group and *n* = 4 in the ingADSC group) by using CellSence imaging analysis software. Vessel density (1 to 100 μm) was manually counted in a given area at the site of injection.

### Statistical analysis

Data are presented as mean ± standard deviation. A two-sided, nonpaired *t* test was used to analyze the flow cytometry and the cumulative doubling data. A Student *t* test with a Welch correction was applied to compare the differentiation capacities of ADSCs isolated from the different tissues. Differences were considered significant at *P* < 0.05. The Prism software package (version 3.0) was used for the statistical analysis.

## Results

### Morphologic and immunophenotypical analysis

**Figure 1:**
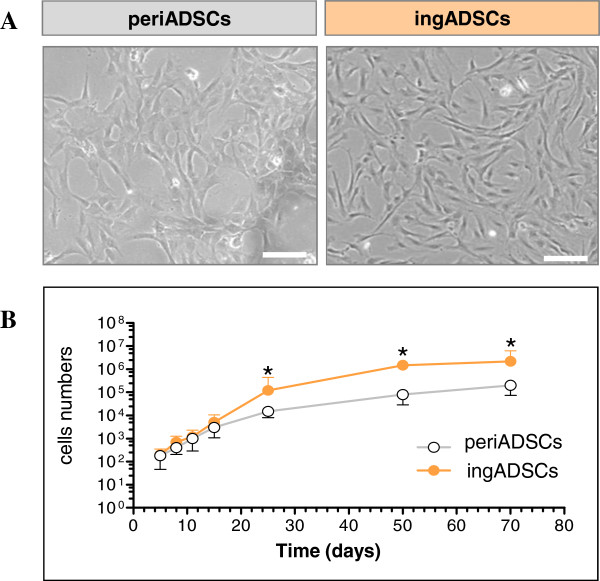
**Characterization of adipose-derived stromal cells (ADSCs) derived from pericardial and inguinal adipose tissue.** ADSCs both from pericardial (periADSCs) and inguinal adipose tissues (ingADSCs) were adhesive and exhibited nondistinguishable spindle morphology that analogous to fibroblasts **(A)**. Both ADSCs exhibited a logarithmic growth in the first 3 weeks; thereafter ingADSCs grew slightly faster than periADSCs, and a significant difference was observed on day 25 (**B**, *n* = 3 in each group). *P < 0.05; bar = 100 μm.

Both pericardial ADSCs (periADSCs) and inguinal ADSCs (ingADSCs) were plastic adherent and, after 2 days of cultivation, they showed microscopically identical morphology with large spreading cells with a highly branched shape and fibroblast-like appearance (Figure 1A). Both ADSCs grew relatively fast, with population-doubling time of 2.3 days under a culture condition of 30% FCS. Every 5 days when 90% confluence was reached, cells were passaged, and total cell numbers were calculated. The growth curve showed that both periADSCs and ingADSCs maintained initially exponential growth, but from 25 days on, ingADSCs still remained in vigorous growth, whereas periADSCs seemingly reached a platform (Figure 1B, *n* = 3 in each group).

Further to characterize whether the two sources of ADSCs are different in surface epitopes, we analyzed the expressions of surface antigens in both ADSCs by using flow cytometry. Both periADSCs and ingADSCs showed positive staining for mesenchymal stem cell markers (CD29, CD44, and CD90) and negative for hematopoietic markers (CD34 and CD45) and an endothelial marker (CD31). Only a slight heterogeneous expression of CD106 was detected between two ADSCs (Table 1), showing a greater CD106-positive subpopulation in periADSCs than in ingADSCs (48.85 ± 13.2% versus 10.22 ± 8.85%; *P* < 0.01; *n* = 4 and 3). Altogether, the immunophenotypical characterization indicated that both periADSCs and ingADSCs shared mostly similar surface epitopes.

**Table 1:** **Flow-cytometric analysis of the immunophenotypes in two ADSC populations**

	periADSCs (*n* = 4)	ingADSCs (*n* = 3)
CD 29	99.90 ± 1.2	99.89 ± 0.1
CD 31	1,12 ± 0	1.48 ± 0.1
CD 34	1.48 ± 1.2	0.68 ± 0.4
CD 44	99.78 ± 1.3	99.84 ± 1.8
CD 45	0.36 ± 0.2	0.43 ± 0.3
CD 90	99.35 ± 0.7	99.85 ± 0
CD 106	48.85 ± 13.2^a^	10.22 ± 8.5
IgG1	1.26 ± 0.8	0.40 ± 0.2

^a^ *P* < 0.01 in comparison with ingADSCs.

### Expression of transcription factors for cardiomyogenesis

**Figure 2:**
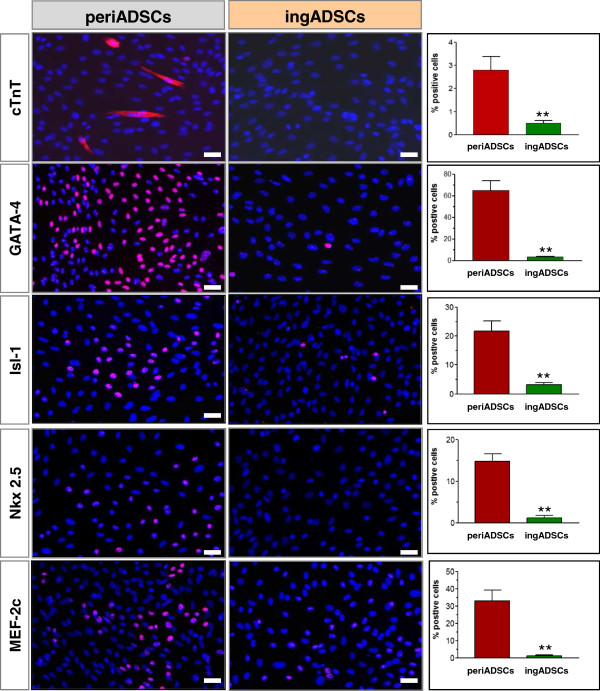
**Immunocytochemical comparison of cardiac factors.** The pericardial ADSCs (periADSCs; *n* = 3) stained partially positive for cTnT, GATA-4, Isl-1, Nkx-2.5, and MEF-2c, whereas the inguinal ADSCs (ingADSCs; *n* = 3) stained almost negative for these factors. **P < 0.01, and bar = 20 μm.

Because periADSCs are associated with the second heart-field development, we further analyzed cardiac-related transcriptional factor with immunostaining. Interestingly, some cTnT-positive cells could already be observed (2.8% ± 1%; *n* = 3) in the first passage of periADSCs before induction to cardiac differentiation, which was never seem in ingADSCs (Figure 2, *n* = 3).

The analysis of the expression of the myogenic transcription factors between the two types of ADSCs showed, to a various extent, expressions of GATA-4, Isl-1, NKx-2.5, and MEF-2c exclusively in the periADSCs. Among them, GATA-4 displayed the highest expression (65.7% ± 19%; *n* = 3), followed by medium expression of Isl-1 and MEF-2c (22.3% ± 11%; *n* = 3 for and Isl-1, 32.3% ± 13%; *n* = 3 for MEF-2c, respectively) and a low level of Nkx-2.5 (14.3% ± 2%; *n* = 3). By contrast, no expression was detected for almost all the factors analyzed, except for a weak expression of Isl-1 in the ingADSCs (3.7% ± 0.5%; *n* = 3). These data suggest that the pericardium-derived ADSCs intrinsically express cardiac factors and thus constitute some biologic properties similar to cardiac progenitor, whereas the ingADSCs of subcutaneous origin are eventually absent.

### Differentiation potentials

**Figure 3:**
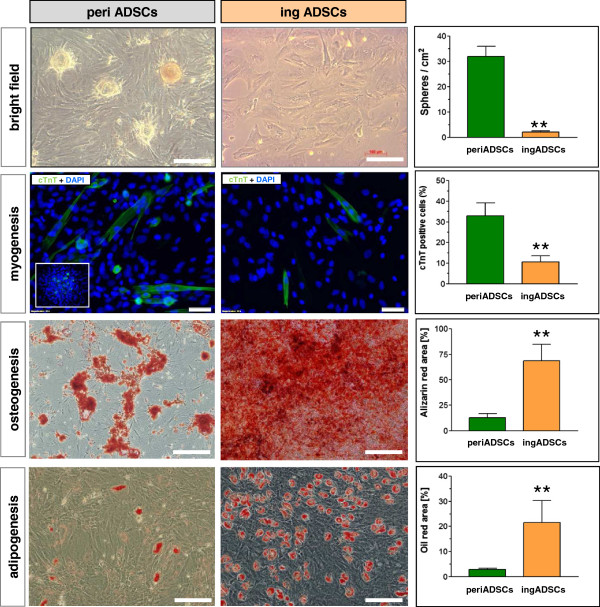
**Comparison of differentiation potential of two sources of ADSCs.** After cardiac induction, both ADSCs showed enlarged morphology, but the pericardial ADSCs (periADSCs) formed spherical structures similar to cardiospheres and significantly more cTnT-expression cells in comparison with inguinal ADSCs (ingADSCs, *n* = 8 in each group). The inset shows the formation of cTnT-expressing cells within the spherical structure. In contrast, periADSCs were less competent to adipogenic (*P* < 0.01; *n* = 5 in each group) and osteogenic differentiation (*P* < 0.01; *n* = 5 in each group), as indicated by oil red and alizarin staining. Bar = 100 μm.

Because periADSCs showed transcriptional potentials for myogenic differentiation, we next compared the potential of two types of ADSCs toward cardiac differentiation. After pharmacologic induction for 7 days, both periADSCs and ingADSCs displayed an enlarged cellular volume but identical morphology. However, significantly more spherical structures analogous to cardiospheres formed in periADSCs in comparison with ingADSCs (32 ± 7 versus 7 ± 1; *P* < 0.01; *n* = 8 in each group; Figure 3). Interestingly, multiple cTnT-expressing progenitors were generated from inside the spheres (insert in Figure 3). These cTnT^+^ progenitors were round but elongated after having migrated out from the spheres. By quantification, the total of the cTnT^+^ cells in periADSCs after induction was significantly more than that in ingADSCs (32% ± 11% versus 11% ± 5%; *P* < 0.01; *n* = 8 in each group), suggesting that periADSCs are prone to cardiac differentiation at given optimal conditions. Conversely, periADSCs were less potent toward osteogenic (*P* < 0.01; *n* = 5 in each group, alizarin staining) and adipogenic differentiation (*P* < 0.01; *n* = 4 in each group, oil red staining) in comparison with ingADSCs after 2-week induction (Figure 3).

### *In vivo*comparison of regenerative activity

**Figure 4:**
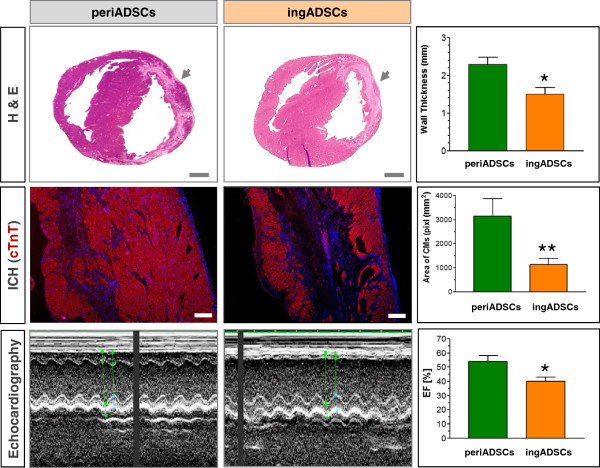
***In vivo*** **comparison of regenerative activity and cardiac function after treatment with two sources of ADSCs.** Rat pericardial (periADSCs) and inguinal ADSCs (ingADSCs) were intramyocardially injected into the infarcted heart, directly after injury. H&E staining demonstrated a significant increase of the wall thickness at the site where periADSCs were injected in comparison with that of ingADSCs 28 days after transplantation (upper panel, *P* < 0.01; *n* = 5 and 4). The site of periADSC injection comprised significantly more cardiomyocytes, suggesting a cardiogenesis induced by periADSCs injection (middle panel, *P* < 0.01; *n* = 5 and 4). Echocardiographic analysis revealed a restoration of contractile activity in the infarcted anterior wall and improvement of ejection fraction (EF) in periADSCs-treated heart (*P* < 0.05, *n* = 5 and 4). **P < 0.01 and *P < 0.05. Bar = 2 mm in the upper and 200 μm in middle panel.

Given that periADSCs showed significant potentials for myogenic differentiation, we performed allograft transplantation of the two types of ADSCs into the infarcted heart to compare their regenerative activity and functional improvement *in vivo*. At 28 days after intramyocardial injection, the ventricular wall at the site that received periADSCs (Trypan blue marked) was significantly thickened in comparison with the wall that received ingADSCs injection (*P* < 0.01 in upper panel of Figure 4, *n* = 5 and 4). Interestingly, the increased wall consisted of many mature cardiomyocytes that stained positive for cardiac cTnT, whereas the ingADSCs-injected site showed significantly fewer cardiomyocytes, mainly located at the endo- and subepicardial territories (middle panel in Figure 4; *n* = 5 and 4).

Most important, the periADSCs-induced structural repair was found to translate into functional amelioration after myocardial injury. Echocardiographic analysis showed a restoration of the thickness and contractile motion of the infarcted ventricular wall and a significant improvement of cardiac ejection fraction (EF) in rats after they were treated with periADSCs in comparison to those after ingADSCs (lower panel in Figure 4 and Table 2; *n* = 5 and 4). Thus, this result suggests that periADSCs generated better regenerative effects *in vivo* characterized by the robust formation of cardiomyocytes and thus enhance structural repair, whereas ingADSCs exhibited only a minor effect.

**Table 2:** **Comparison of cardiac function in the infarcted rats received two population of ADSC treatment**

	periADSCs (*n* = 5)	ingADSCs (*n* = 4)	*P*value
IVSd (cm)	0.18 ± 0.01	0.16 ± 0.02	0.142
LVIDd (cm)	0.71 ± 0.09	0.80 ± 0.06	0.123
LVPWd (cm)	0.26 ± 0.04	0.29 ± 0.04	0.306
IVSs (cm)	0.24 ± 0.03	0.19 ± 0.02	0.024^a^
LVIDs (cm)	0.51 ± 0.07	0.64 ± 0.10	0.08
LVPWs (cm)	0.43 ± 0.05	0.51 ± 0.04	0.037^a^
EDV (μl)	263.8 ± 41.2	344.3 ± 51.4	0.052
ESV (μl)	123.8 ± 38.2	208.5 ± 42.5	0.021^a^
FS (%)	20.1 ± 3.2	28.5 ± 4.5	0.025^a^

^a^ *P* < 0.05 in comparison with ingADSCs.

**Figure 5:**
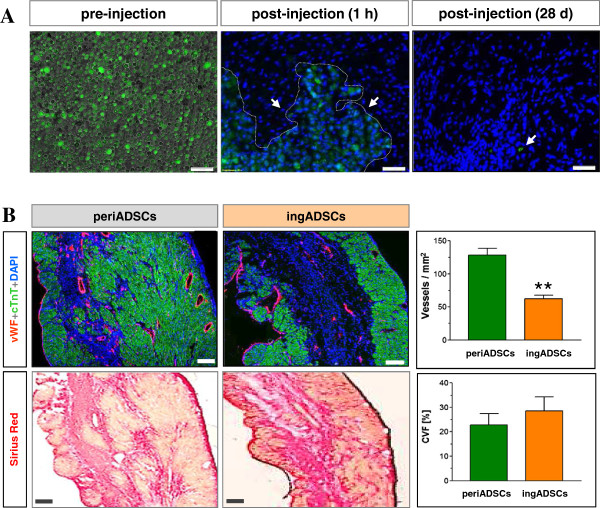
**Cardiac engraftment of periADSCs and the induced vasculogenesis.** The prelabeled periADSCs showed a majority of expression eGFP before injection (**A**, after trypsinization) and optimal cardiac retention (45%; *n* = 3), but only rare eGFP-positive cells were detected in the heart section (<0.01%; *n* = 3). Transplantation of periADSCs increased vessel density at the site of injection in comparison with ingADSCs, indicating proangiogenic effects by periADSCs, but did not alter collagen deposition (**B**, *n* = 5 and 4). **P < 0.01 and bar = 50 μm in A and 200 μm in B.

We further investigated whether the beneficial effects were generated by direct conversion of periADSCs into cardiomyocytes. In this context, periADSCs were genetically labeled by eGFP lentiviral vectors and injected into the infarcted myocardium, as described earlier. Before transplantation, periADSCs showed 81% ± 5% (*n* = 3) eGFP expression and 45% ± 12% (*n* = 3) cardiac retention 1 hour after transplantation (Figure 5A). However, 28 days after injection, only a few GFP-positive cells were detected (<0.01%; Figure 5A, *n* = 3), although in the meantime, a mass of cardiomyocytes formed at the site of injection, suggesting an indirect effect of the engrafted periADSCs on endogenous cardiac progenitors that contributed to cardiac regeneration after periADSCs transplantation.

In addition to striking myogenesis, periADSCs were able to induce angiogenesis when injected into the injured myocardium, as significantly more vWF-positive (endothelial marker) vessel structures were found in the periADSCs-treated heart in comparison with ingADSCs (upper panel in Figure 5B, *n* = 5 and 4). Conversely, the reparative activity of periADSCs seems not to be related to a reduction of cardiac fibrosis, as the collagen volume fraction (CVF) in the hearts receiving either periADSCs or ingADSCs injection showed a similar amount 28 days after myocardial infarction (22.8% ± 8.0% versus 28.50% ± 10.0%, *n* = 5 and 4, lower panel in Figure 5B). Thus, periADSCs rendered structural repair of the injured heart after myocardial ischemia and thus functional improvement by enhancing local myogenesis and vasculogenesis via indirect paracrine effects.

## Discussion

In the present study, we compared the differentiation potential and reparative properties of ADSCs derived from pericardial fat in relation to subcutaneous fat from the groin. Our data suggest that although both ADSCs are phenotypically identical, periADSCs constitute a vigorous potential toward myogenic differentiation by endogenously expressing transcriptional factors for cardiogenesis. Notably, transplantation of periADSCs into the injured myocardium resulted in significant functional improvement and structural repair by robust myogenesis and vasculogenesis. Therefore, pericardial fat-derived ADSCs represent attractive donor cells for the treatment of ischemic heart disease.

In recent years, increasing evidence demonstrated that multipotent stem cells derived from mature adipose tissues are able to differentiate into osteocytes, chondrocytes, adipose cells, cardiomyocytes, and neural cells [3]. Because the adipose tissues are abundant and clinically easy to obtain, they have been considered to be an ideal source for obtaining donor cells in regenerative medicine [11, 12]. In those ongoing preclinical and clinical studies, the ADSCs were mainly isolated from subcutaneous liposuction [13], although visceral adipose tissues also can serve as a source for generating ADSCs. Given that the biology of subcutaneous adipose tissue differs from that of visceral fat in terms of insulin resistance, lipometabolism, and secretion patterns, the subpopulation of the stromal fraction may also vary on where tissue stromal cells are located [14, 15]. For instance, cardiac MSCs have been shown to present cardiovascular-associated features and were more efficient for cardiac repair [16]. However, location-related diversity of ADSCs in terms of differentiation potential and regenerative capacity has not been well documented.

In the present study, we first compared the phonotypic properties of two types of ADSCs from different sources and found that the ADSCs of subcutaneous and visceral origin display the same morphology and surface epitopes that were undistinguishable with flow cytometry (Table 1). Both periADSCs and ingADSCs express mesenchymal markers, as previously reported [6, 17], with only minor heterogeneity of CD106 expression. CD106 is also known as vascular cell adhesion molecule 1 (VCAM-1) and identifies a subpopulation of MSCs with unique immunomodulatory properties [18]. Whether the more sub-fractions of CD106 positive cells in the periADSCs attributed to the in vivo regenerative activity needs to be further characterized.

The striking finding in this study is that periADSCs constitutively express of some key transcriptional factors important for cardiomyogenesis, including GATA-4, Isl-1, Nkx-2.5, and MEF-2c, whereas ingADSCs were fairly absent, indicating that the periADSCs hold a potent intrinsic capability towards cardiac differentiation. This notion was further supported by our inductive experiment, showing the significant formation of cardiospherical structure and the vigorous generation of cTnT-expressing cardiac progenitors in comparison to ingADSCs. Although subcutaneous ADSCs have been well demonstrated to display cardiac potential [1, 2], the more efficient cardiac differentiation of periADSCs may be related to their developmental origin [16]. The epicardial and pericardial MSCs developmentally initiated from the proepicardial cluster of cells located dorsal and adjacent to the heart tube and migrated onto the surface layer to form the second heart field [19] and retained postnatally the ability to form cardiac colony-forming-unit fibroblasts (cCFU-Fs) [9]. Thus, the ADSCs isolated from the pericardial adipose tissue are supposed to constitute some biologic properties similar to cardiac progenitors that contributed to the homeostasis of the heart [9].

The diversity of ADSCs from different locations has been previously described, but their myogenic activity has not been compared [6]. The unique feature of myogenic potency of periADSCs prompts us to investigate further the regenerative capacity relevant to the use of ADSCs for cardiovascular regeneration. In this context, we injected the same amounts of two types of ADSCs into the middle of the infarcted area and analyzed the follow-up structural and functional repair.

Our results demonstrate that, in direct contrast to ingADSCs, periADSCs were able to superiorly induce significant myogenesis and angiogenesis restrictively at the site of injection, but did not alter the infarct size (data not shown) or collagen deposition (Figure 5B). The structural and functional benefits after the transplantation of periADSCs did not result from the direct conversion of the ADSCs into the cardiac lineage, as nearly all prelabeled eGFP-positive periADSCs disappeared 28 days after transplantation (Figure 5A), likely because of apoptosis-mediated cell loss [20]. Regardless of the minimal engraftment in the host myocardium, periADSCs exclusively triggered significant formation of cardiomyocytes (Figure 4), whereas ingADSCs failed to do so, suggesting a unique property of periADSCs on the myogenesis via an indirect action on endogenous cardiac stem cells. The mechanistic interlink showing how periADSCs communicated with endogenous stem cells is not clear, but may relate to apoptotic body-mediated exchange of genetic materials (that is, transcriptional factors) via horizontal gene transfer [21] and/or via indirect paracrine-related proangiogenic and immunomodulatory effects, as reported previously by Naftali-Shani *et al.* [6]. Therefore, those data underscore the definition of paracrine factors and the underlying mechanism by which the endogenous regeneration was evoked.

We are aware of several limitations in the present experiments. First, the differences in adipogenesis and osteogenesis of two types of ADSCs were compared only in *in vitro* conditions and must be characterized further in the *in vivo* condition as to cardiac transplantation. Second, cardiac transplantation was made of a single injection in the infarcted area, and in the future, multiple spots of injection in the border zone may be needed to maximize the functional benefits after myocardial injury. Also, the comparison of cardiac function of two types of ADSCs was analyzed only at the end point (28 days after transplantation), whereas the starting baseline data is still missing. Therefore, further study must assess cardiac function at various time points to demonstrate the kinetics of functional recovery after ADSC-based therapy.

Finally, *de novo* formation of cardiomyocytes must be experimentally proved.

## Conclusions

In summary, our results showed that both ADSCs showed similar morphology and cell-surface epitopes, but periADSCs exhibited potent differentiation toward cardiomyocytes, which could be explained by endogenous expression of important transcriptional factors in periADSCs for cardiomyogenesis. Notably, periADSCs induced significant structural repair in the injured heart in comparison with the subcutaneous donors that have been widely used in the present clinical trials. Because pericardial adipose tissue can be obtained easily, periADSCs may represent attractive donor cells in cardiac regenerative medicine.

## Abbreviations

ADSCs: Adipose-derived stromal cells
cTnT: cardiac troponin T
CVF: collagen volume fraction
DMEM: Dulbecco modified Eagle media
EDV: end-diastolic volume
ESV: end-systolic volume
FCS: fetal calf serum
FS: fractional shortening
GATA-4: GATA-binding protein 4
Isl-1: Isl LIM homeobox 1
IVSd: intraventricular septal (diastole)
IVSd: intraventricular septal (systole)
LVIDd: left ventricular internal dimension (diastole)
LVIDs: left ventricular internal dimension (systole)
LVPWd: left ventricular posterior wall (diastole)
LVPWd: left ventricular posterior wall (systole)
MEF-2c: myocyte enhancer factor 2C
Nkx 2.5: homeobox protein NK-2.5
vWF: von Willebrand factor.
